# Mercy Hospital

**Published:** 1869-09

**Authors:** N. S. Davis


ARTICLE XXXIV.

### MERCY HOSPITAL.

### LAYING OF THE CORNER STONE.

ADDRESS BY DR. N. S. DAVIS.

The ceremonies and impressive exercises connected with the laying of the corner-stone of the Mercy Hospital, took place on the afternoon of July 25, 1869, on the corner of Twenty-sixth Street and Calumet Avenue. A large crowd were in attendance, and, notwithstanding the intense heat, remained and listened with wrapt attention to the addresses, and throughout the whole of the interesting services. While the audience was gathering, and during the time of the laying of the stone, the Immaculate Conception Band, of Father Waldron's church, and Father Conway's Band, of St. James' church, were upon the stand, and added much to the interest of the exercises by their excellent music. The Society of the Immaculate Conception were on the main stand in full uniform.

At 7 o'clock, the procession was formed in the old hospital and marched to the stand, the band meanwhile playing a solemn march. The procession was preceded by six acolytes, each bearing in his hand some holy symbol connected with the services. Following was the Very Rev. T. Halligan, Administrator, who was escorted, with the orator of the day, Dr. N. S. Davis, by the Reverend Fathers McDermott, Conway, Lyon, Scott, Cushman, Leyden, and Waldron, to the platform. The Very Rev. T. Halligan then proceeded to read in Latin the impressive service usual in the Catholic Church on such occasions. In this part of the ceremony he was assisted by the Reverend Fathers already named. At the conclusion of the opening services, architect Boyington placed in the receptacle prepared for it, the box containing the relics and curiosities, after which the stone was lowered to its place. The concluding ceremonies, consisting of blessing the stone and sprinkling it with holy water, were then accomplished, after which the procession reformed and marched back to the hospital.

Dr. Davis was then introduced, and spoke as follows:—

FELLOW CITIZENS:—Until my arrival here, I expected you would be addressed by another, before I was called upon, and lest I should weary your patience, I have prepared to occupy you but a few minutes.

Nineteen years since, the City of Chicago contained less than 30,000 inhabitants; was supplied with only a limited amount of water, by an engine and pump at the foot of Lake Street, and was entirely destitute alike of sewers and public hospitals. The ground on which we now stand was covered with the coarse grass of the unoccupied prairie. The summer previous (1849) that dreaded pestilence, the epidemic cholera, had severely scourged our citizens, and was prevailing more moderately at the time to which I more particularly allude (the summer of 1850). The need of a permanent public hospital had been so far felt that a charter for one had been procured from the State Legislature at a previous session; and the city authorities were making their first attempt at the construction of sewers, by putting down a three-cornered or triangular plank sewer in Clark Street, from the river to Lake Street. It was for the double purpose of protesting against the squandering of public money on such temporary and imperfect structures, and awakening the public attention to the whole subject of the sanitary improvement in our city, that your speaker announced a course of six public lectures on the sanitary condition of the city and the means for its improvement, the proceeds of which were to be used in opening a public hospital under the charter already alluded to. These lectures were given in the old State Street Market Hall, which has long since disappeared, and netted the trifling sum of $100, to which was added some contributions of $5 and $10, from four or five of the more prominent citizens, at the head of whom stood R. K. Swift, then a prosperous banker. With this limited sum of money, *twelve* beds were purchased and put into rooms on the third floor of the south part of the old Lake House, then occupied by a lady as a private boarding-house; and she was engaged to feed and nurse the patients at a specified sum per week. The beds were ready for the reception of patients by the first week of October, 1850, and were soon filled with patients, attended by the late Daniel Brainard as surgeon, and myself as physician. In the spring of 1851, the Trustees named in the charter, consisting of Judges Skinner and Dickey, and Dr. John Evans, finding it not easy to obtain sufficient subscriptions from our citizens to permanently sustain and endow the hospital, were induced to accept an offer made by the Sisters of Mercy, to take charge of it, and meet all the expenses of its management, except the rent of the necessary building. They immediately began to increase the number of beds, and soon occupied one-half of the old Lake House building, the rent of which was paid during the first three years by the contributions of a few citizens, aided by the Faculty of the Rush Medical College. From that time the institution passed entirely into the hands of the Sisters of Mercy, where it has remained till the present time. From the old Lake House, it was removed to a building on Kinzie Street, where it remained only a few months. Then it occupied a building constructed for an orphan asylum, on Wabash Avenue, near Van Buren Street, several years, and from thence it was moved into the building originally constructed for a seminary for young ladies, on the grounds we now occupy. Its growth has been steady and uniform, until, from the small beginning just mentioned, it now lays the foundation of the magnificent structure whose corner-stone you have just placed in its proper position; a structure which will not only remain for ages an ornament to our city, but, what is far better, it will stand as a perpetual monument to the liberality and Christian charity of its founders, and an asylum for the suffering and afflicted of many generations. During its past brief history, without the aid of public appropriations or private endowments, and constantly embarrassed by the temporary structures it has occupied, it has accommodated and kindly treated more than 6000 human beings, suffering from serious diseases, at least one-fourth of whom have been cared for gratuitously. Its doors have been open alike to the afflicted of every class and creed. It has received the professional services, always gratuitous, of the most eminent members of the medical profession, among whom have been Daniel Brainard, Wm. B. Herrick, J. V. Z. Blaney, L. D. Boone, J. E. McGirr, H. A. Johnson, E. Andrews, W. H. Byford, D. T. Nelson, and your speaker. In regard to the ability and faithfulness of the Sisters of Mercy, in the management of the hospital, I can speak in terms of the fullest commendation. Having visited the institution, professionally, almost constantly, from its incipient organization to the present hour, I must say that in cleanliness, good order, kindly attention, and Christian liberality I have not seen them equaled in any other public hospital in this country. With the building now in process of erection, each department of the hospital will be amply provided for. Besides the public wards, there will be many rooms affording every comfort and convenience for the accommodation of such patients as are able to provide themselves with the comforts, as well as the necessaries, of life, and yet need the advantages for special treatment that the hospital affords. Among its inmates heretofore have been patients from all the North-western States, as well as from the City of Chicago. To sustain such an institution, however, requires, in addition to the faithfulness and skill of its immediate attendants, the active aid and sympathy of the whole community. The untiring labors of the Sisters may be bestowed without money and without price; the Medical Faculty may give it the benefit of their highest skill without a fee; but to furnish it with food, fuel, light, water, and medicines requires money, and that, too, in larger quantity than can be derived for the payment of board by the patients. There are many of both sexes having no homes of their own, who, when taken sick, are not proper subjects to be made a county charge, and to whom the well-regulated public hospital affords the only safe resort. If their sickness proves severe, the little money on hand at the beginning is exhausted long before they are well, and they must remain gratuitously or be turned helpless away. There are others whose education, social relations, prejudices, perhaps, are such that they could not be induced to go into the public wards of a hospital for the city or county poor, and yet who are destitute of the means with which to pay for either board or the services of a physician or surgeon, and, yet, who would gladly avail themselves of the advantages of an institution like this, if a way was provided by which they could be admitted. Hence, when this noble structure shall have been finished; when the self sacrificing Sisters of Mercy shall have completed and furnished every room, from basement to attic, with themselves in readiness to care for the sick, and the board of physicians and surgeons ready to aid them, there will yet lack one thing to render its means of usefulness complete.

To supply this one thing, and make the Mercy Hospital of Chicago not only one of the best hospitals, but also one of the *noblest charities* in the world, it should receive such a pecuniary endowment as would make, at least, twenty of its beds perpetually free for the occupancy of those who are destitute as well as sick. *Twenty* beds thus free would accommodate an average o

150 patients per annum. Who can estimate the benefits that would result or the amount of human suffering that would be relieved in the lifetime of a single generation? And are there not twenty men within hearing of my voice who could each donate $1000, to be invested and held in trust, the interest of which would secure twenty free beds for suffering humanity? Is there any other way in which a like sum could be invested with a certainty of securing an equal amount of good?

To clothe the naked, to feed the hungry, to provide for and heal the sick are among the highest and most sacred injunctions of the Divine Author of Christianity.

However much the world of mankind may be divided in reference to religious creeds and ceremonies, there can be but one sentiment in regard to the universally binding character of these injunctions. They are broad in their scope as the brotherhood of man, and as binding as the divine impress can make them. Then, let every thoughtful man who has an abundance of this world's goods, reflect that for every dollar he will be called to render an account. Not as to whether he obtained it honestly or by fraud; not whether he expended it for the gratification of his pride or passions, or hoarded it in his safe, but in that great day of fina judgment, we are told the question will come, did ye clothe the naked; did ye feed the hungry; did ye visit the prisoner; did ye minister to the sick? Christianity demands of its votaries not negative virtues merely, but positive acts of charity and human kindness.

Then, fellow-citizens, while this noble structure is advancing to completion, let me entreat you to make such provisions, pecuniarly, as will secure to it the highest possible degree of usefulness, and you will thereby secure to yourselves, also, the reward of the Christian, namely, the consciousness of having added to the sum of human life and happiness.

After the public exercises the distinguished guests present were invited to an elegant collation, prepared by the Sisters, in the parlors of the old hospital. There were present beside the Very Rev. Father Halligan and the Reverend Fathers already mentioned, the orator of the day, Dr. Davis, the architect, Mr.

Boyington, Dr. Byford, Dr. Andrews, Dr. Johnson, and a number of others who, during the past years, have connected themselves prominently, by their charitable acts, with the institution. After doing justice to the viands prepared, the pleasant company enjoyed a social hour, and were invited to inspect the building and the proposed improvements.

The plans for the new hospital were made from suggestions that were offered by Dr. Edmun Andrews, who has spent much time abroad studying the construction and architecture of the most noted European hospitals. This hospital will contain every improvement, and be one of the most complete and perfect in this country.

The following is a description of the hospital as it will be when completed:—The principal front of the building is on Calumet Avenue, occupying 200 feet, and the average depth of the main building is 35 feet. On 26th Street, the frontage is 86 feet, which forms the south wing of the building. The north wing is formed by the old hospital, which has been greatly enlarged and remodeled throughout, to make it correspond with the other portions of the structure. There is a third wing immediately in the rear of the centre of the main building which extends back 86 feet, thus making the three wings of an equal size and depth.

The building is to be constructed in the Byzantine style of architecture, and will be three stories high, with a basement which is partly above the ground, and, therefore, might be considered another story, as it is nine feet in the clear. Beneath the whole is a deep cellar, and over all a large, roomy attic, which may be used in case of need for rooms. The front of the building is divided into five sections, two of which recede in order to give place to the towers which are placed at each of the corners. These towers have a canopy top, and as they will be used as ventilators, the whole building will be furnished with pure air in abundance.

In the centre of the building is the main entrance to the basement, which opens into a hall that leads back to the corridor. This corridor passes along the rear wall of the building, on each of the three stories and basement. This a new method of constructing the interior of a hospital, it hitherto having been the plan to place the corridor in the centre of the building, with the rooms on either side opening into it. After a careful study of many plans, it is believed, however, that the present one is much more suitable for the purposes for which the building is being erected, as the air is thus allowed to circulate freely through the rooms from the front and rear. Thus a continuous circulation of pure air is secured, while the foul air escapes through the rear windows, instead of passing through the rooms on the opposite side of the corridor. By this plan of ventilation, infection from contagious disease is rendered almost impossible. As you enter the main hall in the basement, on one side is the visitors' room, while upon the other side, directly opposite, is the reception room for patients. In the rear of these, on the south side of the main entrance, is a pleasant roomy dining-hall, 24 by 37 feet; back of this is the kitchen, 25 by 25½ feet, while still back of these rooms are the store-room and pantry, six cells for uncontrollable or quarrelsome patients, and a ward 28 feet wide by 38 feet long. On the north side, the whole of this floor is divided up into rooms, 15 by 20 feet, which are to be used by the patients who are sent to the hospital for treatment. The laundry department is also on this floor, in the centre wing, and is to be fitted up with every convenience, and provided with all the modern inventions for the washing, drying, and ironing of clothes.

There are three entrances, which lead up by three flights of steps to the main floor. As in the basement, the hall runs directly back to the corridor in the rear, while one side is a reception room for those who come to visit the institution; while on the opposite side is the room where the patients are to be examined. A large hall, 30 feet wide by 45 long, in which medical and other lectures are to be delivered, is on this floor, underneath the chapel. Seats are arranged in the hall that will comfortably accommodate 300 people. In the rear of the hall is an octagonal recess for the lecturer, and above is a large window, or skylight, that throws a flood of light down upon the lecture-desk. The hall in front is finished with a row of cases, in which the medicines are to be kept.

The north wing of this main floor is used wholly for patients' rooms. Wards, 25 feet wide by 37 feet long, are situated north and south of the main hall, on the corridor. In the south wing there is likewise a large ward 25 by 80 feet, while between the corridor and the south wing is the nurses' room, 12 by 16 feet. Adjoining are water-closets, a large bath-room, which all the patients are allowed to use, a dumb waiter which carries the food up to all the floors, and all the other conveniences that are needed to care properly for the patients. The height of the ceiling on this floor is 18 feet, giving the room an airy, light appearance.

In the second story the halls and corridors are the same as those below, and the ceiling is 13 feet high. In the south wing of this floor a number of pleasant parlors, 21 feet long by 11 wide, are arranged, in which the convalescent patient may exercise and enjoy social intercourse. In addition to these is a ward 25 by 30 feet. There are likewise water-closets—one in each section of the building—and a bath-room. There are also two suits of rooms, into each of which passes the dumb waiter. The remainder of this floor is divided up into separate compartments, of the same size as these below, for the accommodation of the patients.

The ceiling on the third story is also 13 feet high, and the whole floor is laid out in about the same manner as the floor below, with the exception of the chapel. This is finished in a neat and tasteful style, being plain but convenient in its construction. A small gallery is erected in the front end of the chapel, while a large chancel, with all the accessory furniture, is situated in the rear. The seating capacity of this chapel is about 300, and is comfortable.

The building is covered by a large attic, which is both well ventilated and lighted, and may be used for various purposes, should the accommodations below at any time prove insufficient. In the large cellar underneath the building is placed the furnace, fuel-room, and all the necessary appliances for heating the building, which is done by steam. Beside the steam apparatus, nearly all the rooms are furnished with grates for coal fires, to be used in cases of emergency.

In addition to all these conveniences, the hospital is provided with a large elevator, which runs from the basement to the upper story. This has been constructed so that patients may be removed from one part of the building to another, without removing them from their cots; the elevator having been made large enough to carry a full-sized cot. A spiral staircase surrounds the elevator and connects all the floors of the building. There is also, in addition to this and the main stairway, an additional one in each wing of the building. The hospital is finished throughout in pine, grained to resemble oak.

The roof has a steep pitch, and is covered with slate, with three pediments, one in front of each wing.

The building will cost, when completed, about $60,000.

